# Biochemical and structural characterization of a novel ubiquitin-conjugating enzyme E2 from *Agrocybe aegeria* reveals Ube2w family-specific properties

**DOI:** 10.1038/srep16056

**Published:** 2015-11-03

**Authors:** Chao Qi, De-Feng Li, Lei Feng, Yanjie Hou, Hui Sun, Da-Cheng Wang, Wei Liu

**Affiliations:** 1 National Laboratory of Biomacromolecules, Institute of Biophysics, Chinese Academy of Sciences, Beijing, 100101, China; 2 University of Chinese Academy of Sciences, Beijing, 100049, China; 3 The College of Life Sciences, Wuhan University, Wuhan, Hubei Province, 430072, China; 4 Institute of Immunology, The Third Military Medical University, Chongqing, 400038, China

## Abstract

Ubiquitination is a post-translational modification that is involved in myriad cellar regulation and disease pathways. The ubiquitin-conjugating enzyme (E2) is an important player in the ubiquitin transfer pathway. Although many E2 structures are available, not all E2 families have known structures, and three-dimensional structures from fungal organisms other than yeast are lacking. We report here the crystal structure of UbcA1, which is a novel ubiquitin-conjugating enzyme identified from the edible and medicinal mushroom *Agrocybe aegerita* and displays potential antitumor properties. The protein belongs to the Ube2w family and shows similar biochemical characteristics to human Ube2w, including monomer-dimer equilibrium in solution, α-NH_2_ ubiquitin-transfer activity and a mechanism to recognize backbone atoms of intrinsically disordered N-termini in substrates. Its structure displays a unique C-terminal conformation with an orientation of helix α3 that is completely different from the reported E2 structures but similar to a recently reported NMR ensemble of Ube2w. A mutagenesis study on this novel enzyme revealed that an intact C-terminus is significant for protein dimerization and enzymatic activity. As the first crystallized full-length protein of this family, UbcA1 may supersede the truncated X-ray structure of Ube2w (PDB entry 2A7L) as the representative structure of the Ube2w family.

The ubiquitin-proteasome pathway (UPP) is a highly selective proteolytic mechanism that plays crucial roles in protein quality control, cell cycle control, proliferation, development, signal transduction, transcriptional regulation, receptor down-regulation, and synaptic plasticity^1^,^2^,^3^,^4^. The UPP begins with covalent attachment of a ubiquitin (Ub), which is a highly conserved 76-amino-acid polypeptide, to a substrate protein^5^. Protein ubiquitination is a multistep process orchestrated by three enzymes: a Ub-activating (E1) enzyme, a Ub-conjugating (E2) enzyme and a Ub ligase (E3). E1 activates ubiquitin by forming a high-energy thioester linkage between its catalytic Cys residue and the C-terminal glycine of Ub. Next, E2 transfers the activated Ub via a ubiquitin thioester intermediate to a substrate specifically bound to E3, which catalyzes the formation of an isopeptide bond between the C-terminus of ubiquitin and the ε-NH_2_ group of a lysine on the substrate. Subsequently, the E2–E3 pair often switches to polyubiquitination by cyclically attaching additional Ub molecules to one of the Lys residues in the substrate-linked ubiquitin. Despite the common Ub attachment to the ε-NH_2_ side chain of Lys residues in the ubiquitinated protein, certain E2s may transfer Ub to noncanonical amino acids such as cysteine, serine and threonine or even to α-NH_2_ of some substrates^6^,^7^. Recent studies from different laboratories have identified Ube2w as the first known N-terminus modifying E2^8^,^9^,^10^.

E2s that are encoded in various genomes constitute a huge protein superfamily^11^,^12^,^13^. All E2 enzymes are characterized by the presence of a highly conserved ubiquitin-conjugating (Ubc) domain comprising ~150 amino acids and adopting a similar overall fold^14^. E2s that contain variable N- or C-terminal extensions appended to the Ubc domain are more common, and the E2 superfamily is accordingly divided into four classes: class I, Ubc domain only; class II, Ubc plus a C-terminal extension; class III, Ubc plus an N-terminal extension; class IV, Ubc plus both N- and C-terminal extensions^11^,^12^. Three or four α-helices, an anti-parallel β-sheet formed by four strands, and a short 3_10_-helix compose the structural core of the Ubc domain. The N-terminal helix and two loops, L4 and L7 (also referred to as L1 and L2 in some literatures), form the interaction site with E1 and E3^15^,^16^. Critical mechanical elements include the active site cysteine nestled in a shallow groove close to the 3_10_-helix, an upstream ‘HPN’ tripeptide and a downstream highly conserved tryptophan that often contacts a conserved proline residue located on L4^11^,^13^. In addition to these common features, the Ubc fold represents a rare example in enzyme evolution with high structural diversity^11^, and even the Ubc domain alone can be classified into 17 subfamilies^12^. Multiple lines of evidence have demonstrated that some physiological functions and cellular activities of E2s are related to family-specific structural elements^11^,^16^. To date, 160 structures classified in the CATH ubiquitin-conjugate enzyme superfamily (3.10.110.10) have been deposited in the Protein Data Bank (PDB), most of which are of an individual Ubc domain. Despite the abundance of available structures, not all of the E2 families defined by Michellel *et al.*^12^ have a known structure, and no crystal structure from fungi other than yeast has been reported. Aside from the generalized motifs, studies on family-specific structural features and the relevant mechanistic roles were relatively insufficient. All of these facts have encouraged researchers to determine more E2 structures with unrelieved enthusiasm.

Mushrooms have been recognized for their unique medicinal application for over five millennia^17^,^18^. *Agrocybe aegerita*, an edible mushroom with wide-range growth, was found to have potential antitumor properties^19^,^20^. A gene encoding a putative Ub-conjugating enzyme was fished by screening its cDNA library in a recent transcriptomic analysis^21^,^22^. The gene product comprising 152 amino acids showed considerable sequence similarity to E2s in the Ube2w family. Subsequent biochemical assay confirmed that the protein possesses definite Ub-conjugating activity, and it was named UbcA1. We describe here the biochemical and structural characterization of this novel Ub-conjugating enzyme, which is the first fungal E2 enzyme other than the homologues from yeast crystallized to date.

## Results

### UbcA1 belongs to the Ube2w family

**Figure 1:**
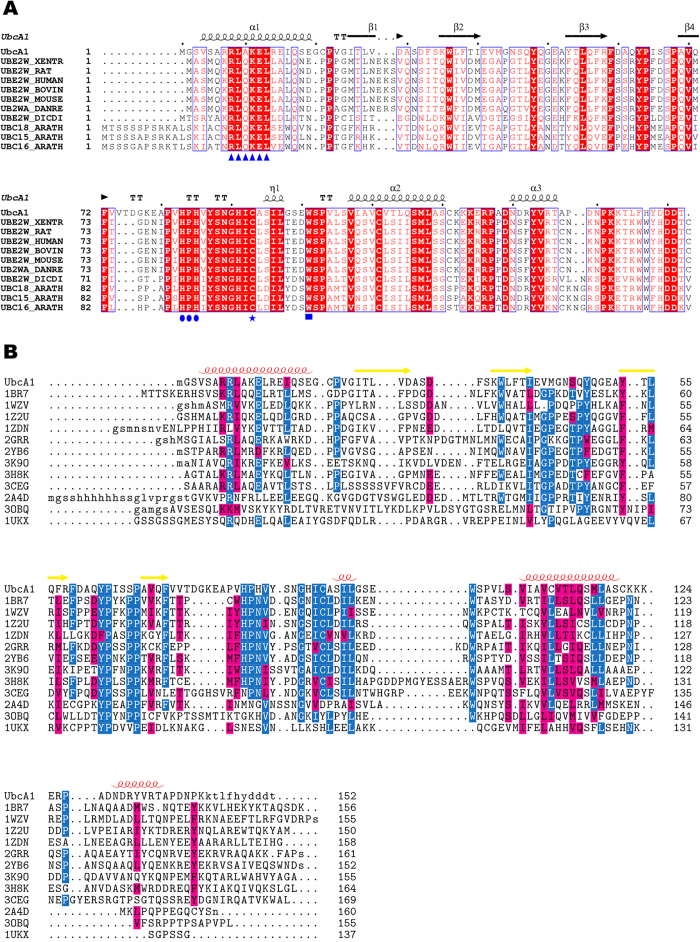
Sequence comparison reveals that UbcA1 belongs to the Ube2w family. (**A**) Sequence alignment of UbcA1 and Ube2w homologues. The catalytic residues and conserved motifs are highlighted by blue symbols on the bottom line. (**B**) Structure-based sequence alignment between UbcA1 and 12 representative domains from the Ub-conjugating enzyme superfamily (3.10.110.10) defined in the CATH database^28^, with PDB codes of 1BR7, 1WZV, 1Z2U, 1ZDN, 2GRR, 2YB6, 3K9O, 3H8K, 3CEG, 2A4D, 3OBQ and 1UKX. The secondary structures of UbcA1 are given on the top line in both panels.

The ORF of UbcA1 fished from a cDNA library of fruiting body of medicinal fungus *Agrocybe aegerita* encoded a 152-amino-acid sequence showing highest similarity with Ube2w enzymes (50% identity with human Ube2w) and *Arabidopsis thaliana* Ubc15, 16 and 18 (Fig. 1A), and considerably lower similarity with E2s from other families (Fig. 1B). The protein contained an individual Ubc domain without an N- or C-terminal extension. All known conserved elements throughout the E2 superfamily were present in its sequence, including a catalytic cysteine at position 93 and a downstream tryptophan (residue 101). The upstream HPN signature (residues 83–85), however, was replaced by HPH in UbcA1, which is a unique property of the Ube2w family^11^,^12^. Another generalized motif, xR*ϕ*xx-x (x, any residue; *ϕ*, hydrophobic; -, negative) close to the N-terminus^13^ appeared in the sequence of UbcA1 and Ube2w homologues as a variant version of RR*ϕ*xKE (Fig. 1A).

### Ubiquitin is attached to the N-terminal α-NH_2_ group of UbcA1 *in vitro*

**Figure 2:**
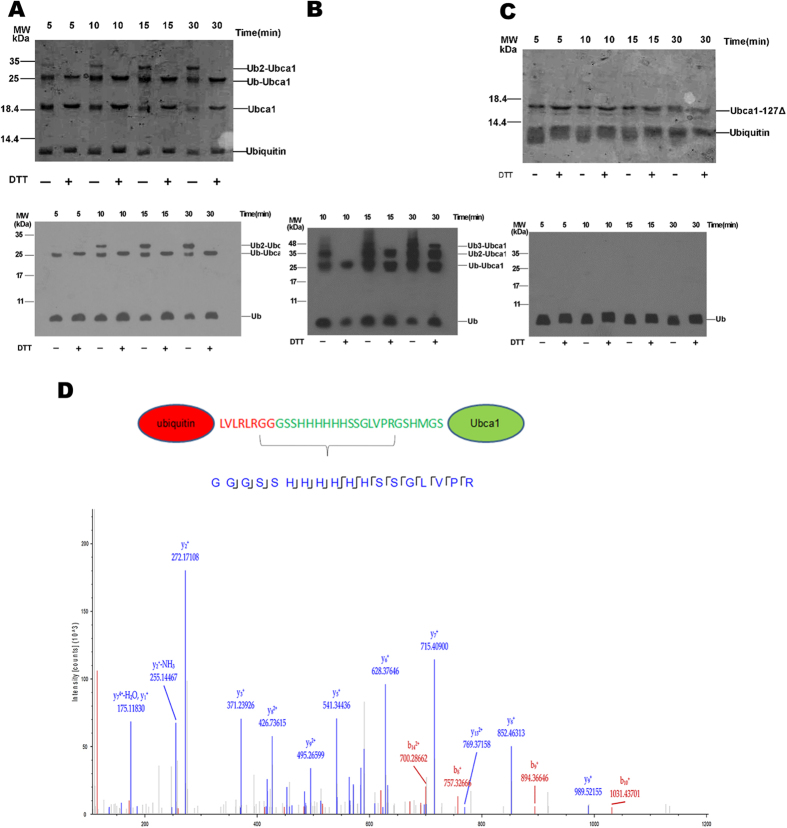
UbcA1 conjugates ubiquitin to the N-terminus of itself in the absence of an E3. (**A**) Single Ub-transfer to the α-NH_2_ group and thioester formation shown on a Coomassie-stained SDS gel (Upper panel) and a western blot gel for Ub (Lower panel). UbcA1 was incubated with E1 and Ub for 5, 10, 15 or 30 min prior to the addition of SDS sample buffer with or without DTT, as indicated beneath the gel. (**B**) Multiple Ub-transfer to the N-terminus when UbcA1 is incubated with HA-Ub rather than wild-type Ub. (**C**) UbcA1-127Δ (removal of residues downstream of 127) could not conjugate Ub to itself, indicating that an intact C-terminus is required for the enzymatic activity, as shown on the Coomassie-stained SDS gel (Upper panel) and a Western blotted gel for Ub (Lower panel). (**D**) Identification of N-terminally ubiquitinated UbcA1 by LC MS/MS analysis after excision of protein band from a silver-stained SDS-polyacrylamide gel and in-gel tryptic digestion.

Because all family-specific signatures were present in the UbcA1 sequence, we wondered whether this novel E2 enzyme had similar Ub-transfer activity to human Ube2w. Ube2w mediates Ub-attachment to the N-terminus in a wide range of substrate proteins, including itself, and such modification can even be done in the absence of an E3, though at a substantially slower rate^8^,^9^,^10^. As downstream E3s and possible substrates in *Agrocybe aegeria* are all unknown, we performed an E1-dependent Ub-loading assay to detect Ub-modification to UbcA1 itself. A mono-Ub conjugate was observed 5 min after incubation with a human E1 enzyme and excess ubiquitin, and a di-Ub conjugate was detected 10 min after the reaction started, indicating that more than one Ub molecule could be loaded onto UbcA1 *in vitro*. Because Ube2w only mediates mono-ubiquitination *in vivo*^23^,^24^, we supposed that one Ub was loaded to the α-amino group of UbcA1, while the other conjugated to the catalytic Cys residue through a thioester bond. To test this possibility, the reaction solution was incubated with SDS sample buffer containing 0 or 200 mM DTT before loading onto an SDS gel. The band corresponding to the di-Ub conjugate completely disappeared in the presence of DTT on either a Coomassie-blue-stained SDS gel or a western blot (Fig. 2A), which confirmed our speculation.

Vittal *et al.* reported that Ube2w can recognize backbone atoms of intrinsically disordered N-termini in its substrates, allowing it to mediate multiple addition of Ubs harboring a 13-amino-acid N-terminal human influenza hemagglutinin (HA) tag to the substrate^10^. To detect whether UbcA1 has a similar activity, we repeated the *in vitro* Ub-loading assay using HA-Ub instead of wild-type Ub, and observed poly-Ub attachment to the N-terminus of itself (Fig. 2B). This result clearly indicates that UbcA1 relies on the same mechanism as Ube2w to mediate α-NH_2_ ubiquitination.

Finally, to confirm the N-terminal Ub-conjugation, mono-ubiquitinated UbcA1 was purified from an excised band of a reduced silver-stained SDS gel and subjected to mass spectrometry (MS) before in-gel tryptic digestion. The resulting LC MS/MS spectra demonstrated the presence of a peptide consistent with N-terminally ubiquitinated UbcA1 (Fig. 2D).

### Dimeric structure of UbcA1 in the crystal

**Figure 3:**
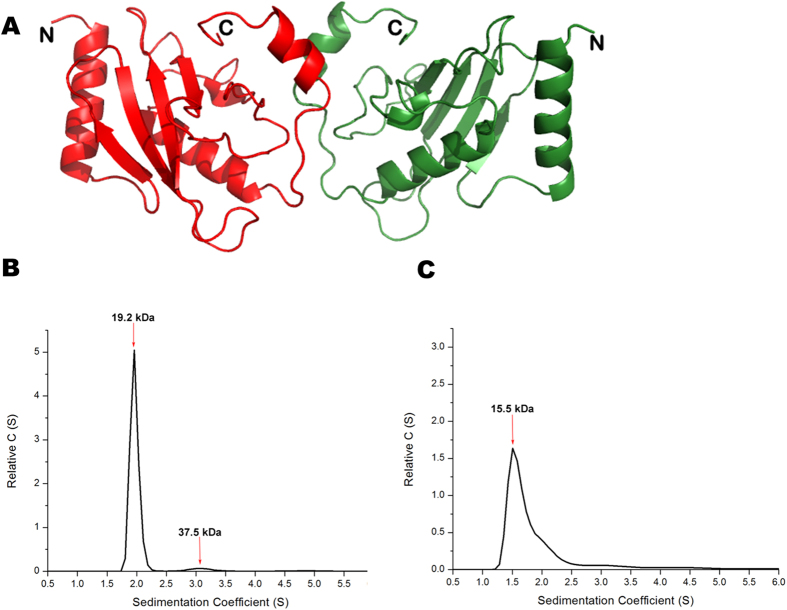
The dimeric structure and the monomer-dimer equilibrium of UbcA1. (**A**) Ribbon diagram showing a dimeric architecture in the crystal structure. (**B**,**C**) Velocity sedimentation distribution of wild-type UbcA1 (**B**) and UbcA1-127Δ (**C**). The molecular weight estimated from each signal is given on the top of corresponding peak.

UbcA1 was crystallized after protein purification. The structure was determined by means of molecular replacement and refined at 1.7 Å. The asymmetric unit in space group *C2* contained two monomers related by a two-fold non-crystallographic axis (Fig. 3A). The refined model comprised 282 amino acids (residues 2 to 142 in both subunits) and 320 solvent molecules. The N-terminal His-tag and the C-terminal sequence from residue 143 to 152 were poorly defined due to lack of traceable electron density and had to be omitted from the model. The crystallographic and stereochemical quality of the final model was acceptable (Table 1).

The two protein protomers sitting in the asymmetric unit formed a dimeric architecture with overall dimensions of 50 Å × 50 Å × 80 Å. By comparison with most dimeric proteins, however, the UbcA1 dimer looked rather uncompact and somewhat unstable, which seemed to be attributable to a small buried area upon dimer formation. Each subunit contributed only 6% (468 Å^2^ vs. 7754 Å^2^) of the total solvent-accessible surface at the dimer interface, and the protein relied almost entirely on a short C-terminal helix (α3) to form subunit-subunit interactions (Fig. 3A).

### Monomer-dimer equilibrium of UbcA1 in solution

Despite the dimeric architecture revealed in the crystal, we were not sure whether this was the real state of UbcA1 in solution because the majority of E2s function as monomers. The purified protein was subjected to velocity sedimentation assay using a Beckman XL-I analytical ultracentrifuge. Two sedimentation signals corresponding to UbcA1 monomer and dimer were displayed on the sedimentation coefficient distribution curve (Fig. 3B), indicating this novel E2 exists in a monomer-dimer equilibrium, which is another property similar to human Ube2w^25^. It is notable, however, that the equilibrium in UbcA1 seemed quantitatively different from Ube2w because the dimer signal was much lower than the monomer signal (monomer : dimer = 32.65:1), indicating the monomer was the dominant form in solution.

Taken together, all biochemical characteristics of UbcA1 revealed in our experiments, including the α-NH_2_ ubiquitination and the monomer-dimer equilibrium as well as the catalytic mechanism of recognizing intrinsic disordered backbone atoms of substrates, were comparable to Ube2w^10^,^25^, which suggests that these features are the common properties of this protein family.

### Subunit structure and catalytic elements

**Figure 4:**
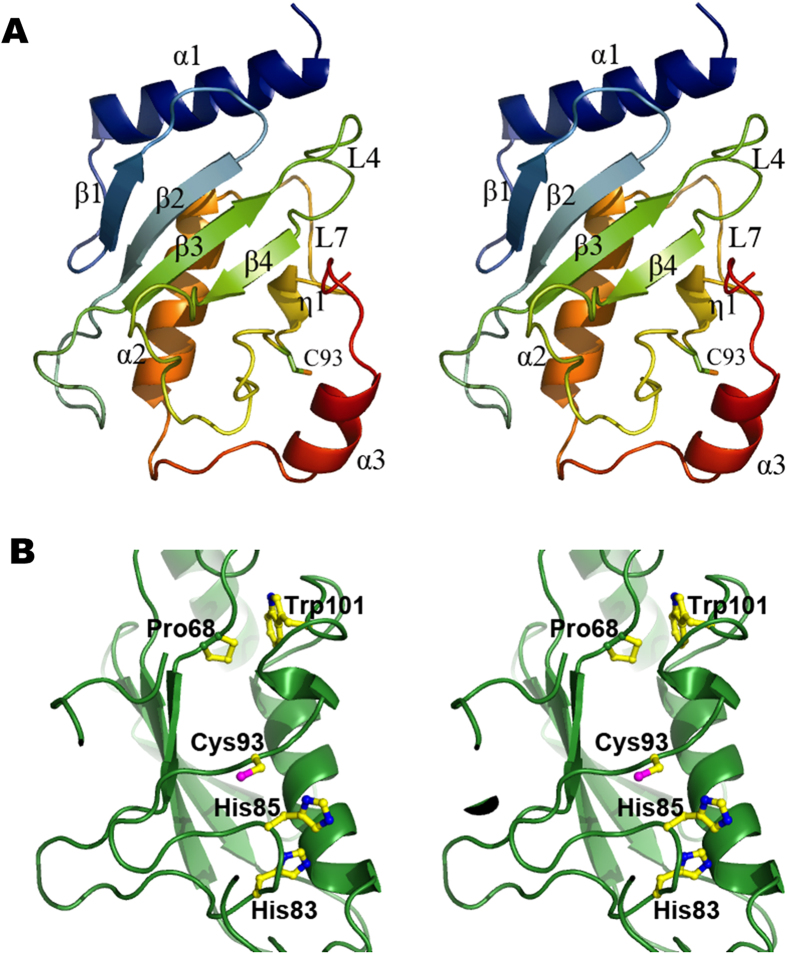
Subunit structure of UbcA1 in the crystal. (**A**) Stereo view of the subunit structure resembling the canonical Ubc fold. The protein model is rainbow-colored, from the N-terminus in blue to the C-terminus in red. (**B**) Conserved amino acids recognized as the key catalytic elements. All these residues are represented by a stick-ball model with atom-indexed colors: carbon, yellow; oxygen, red; nitrogen, blue; sulfur, magenta.

The subunit structure of UbcA1 showed a typical elliptical scaffold that was basically same as the generalized topology of E2s^11^. The structural core was composed of a four-stranded antiparallel curled β-sheet flanked by a C-terminal flap-like structure containing a short 3_10_ helix near the active site. Extending from it, there were an N-terminal α-helix (α1) and two helices (α2 and α3) close to the C-terminus (Fig. 4A). Two loops, L4 bridging β3 and β4 and L7 connecting the 3_10_ helix and α2, were present on one side of the β-meander. These loops, together with α1, are believed to compose the interaction surface for E1/E3 binding^15^,^16^.

The architecture of the active site in UbcA1 was consistent with canonical eukaryotic E2s. The catalytic cysteine (Cys93) was located in the long loop region following the β-meander, two residues upstream of the 3_10_-helix, with its side chain exposed on protein surface (Fig. 4B). As a family-specific feature, the side chains of the two upstream conserved histidine residues (His83 and His85) occupied similar positions to their counterparts of the HPN motif in classical eukaryotic E2s other than the Ube2w family (Fig. 4B). Such a spatial arrangement allows the second histidine (His85) to play a similar role to asparagine to serve as a stabilizer for an oxyanion hole formed in the process of enzyme turnover^26^. The highly conserved tryptophan, Trp101, was located three positions downstream of the 3_10_-helix, with its side chains hydrophobically interacting with a conserved proline residue (Pro68) on L4. This pair of residues was believed to play an important structural role in stabilizing the E2 fold^11^.

### Structural comparison with human Ube2w

**Figure 5:**
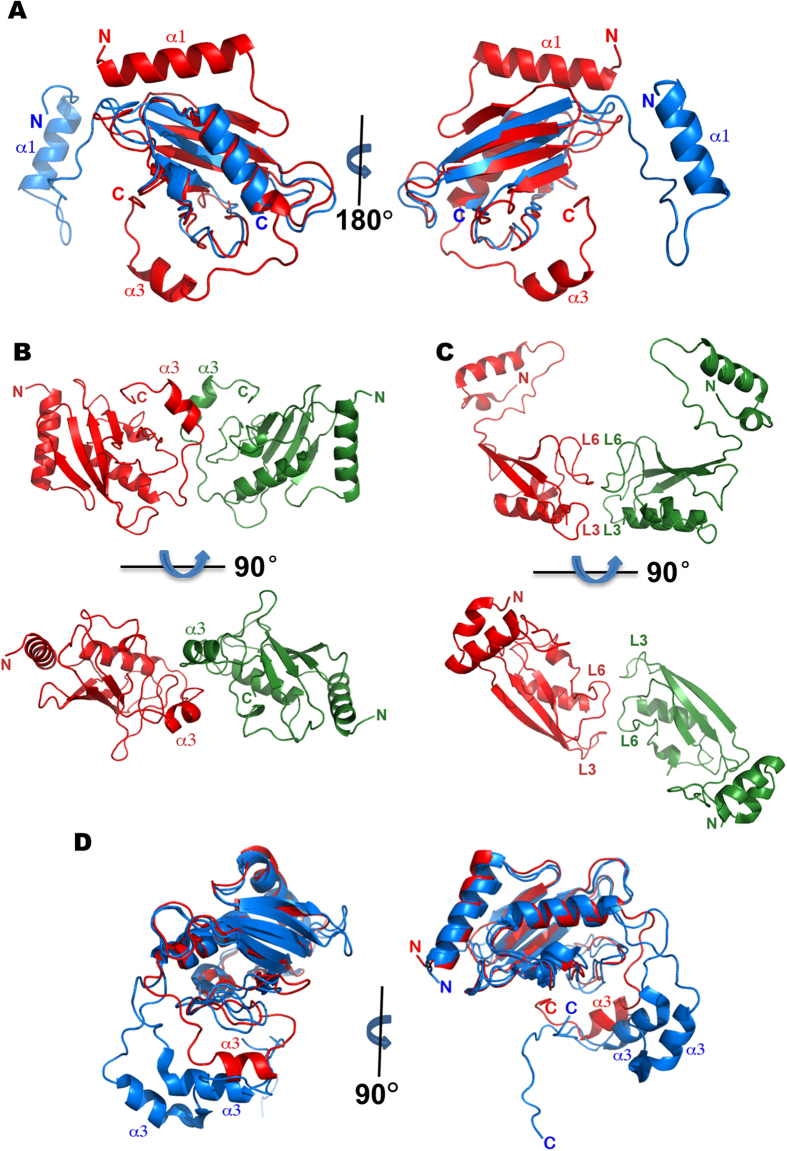
Structural comparison between UbcA1 and human Ube2w. (**A**) Structure superimposition of the UbcA1 structure (red) and the crystal structure of Ube2w with the PDB code of 2A7L (blue) showing inconsistent N-terminal regions (residues 1–32) due to deviation of the latter structure from the canonical Ubc fold. (**B**) The dimeric model of UbcA1 displaying the protein-protein interface at the C-terminal segment and helix α3 in particular. (**C**) The dimeric model of 2A7L showing the interface formed by two loops, L3 and L6. (**D**) Structure superimposition of UbcA1 (red) and a recent NMR model of Ube2w with PDB code 2MT6 (blue). Both adopt the canonical fold except for a C-terminal peptide covering the last helix (α3). For clarity, only two models from the NMR ensemble are shown.

Human Ube2w (PDB entry 2A7L) is the only available X-ray structure in the Ube2w family to date, but unfortunately it was crystallized from a truncated construct lacking the polypeptide after residue 117^27^. Superimposition of UbcA1 onto it revealed considerable structural variance between them. Although structural alignment gave an rmsd of 1.2 Å on 88 C_α_ positions, the N-terminal region (residues 1–32) could not be even slightly overlaid (Fig. 5A). That segment in Ube2w adopts an unusual conformation radically deviating from the canonical Ubc fold. Its N-terminal helix (α1) stretches away from the structural core and the first β-strand (β1) is replaced by a flexible loop, which may render the structure unstable. In another respect, although the two enzymes both form dimers in the crystal, their dimerization modes look completely dissimilar. In contrast to the helix-helix interactions at the dimeric interface of UbcA1 (Fig. 5B), two loops in Ube2w, L3 and L6, mediated dimerization through loop-loop contacts (Fig. 5C). Probably due to the truncated protein used in crystallization, the apparent structural inconsistencies between the human Ube2w structure and the generalized topology of E2s were likely driven by crystal packing and hence might not reflect a physiological conformation.

In the CATH database^28^, the superfamily of ubiquitin-conjugating enzymes (3.10.110.10) currently contains 29 representative domains with lower than 35% sequence identity between any two of them. As the only available structure, 2A7L is the representative model of the Ube2w family (Cluster 3.10.110.10.16.1.1.1). On realizing the possible flaws existing in that structure, we supposed that our model better represented this protein family because UbcA1 was crystallized from a full-length construct and its structure resembled the canonical E2 fold. Notably, an NMR ensemble of human Ube2w (PDB entry 2MT6) was reported recently^10^, which showed a canonical position and orientation of the N-terminal helix (α1) and a disordered C-terminal helix (α3). Superimposition of our structure and this ensemble displayed a perfectly overlaid Ubc domain but a non-overlaid C-terminal peptide following helix α2 (Fig. 5D).

### UbcA1 shows a unique C-terminal conformation

**Figure 6:**
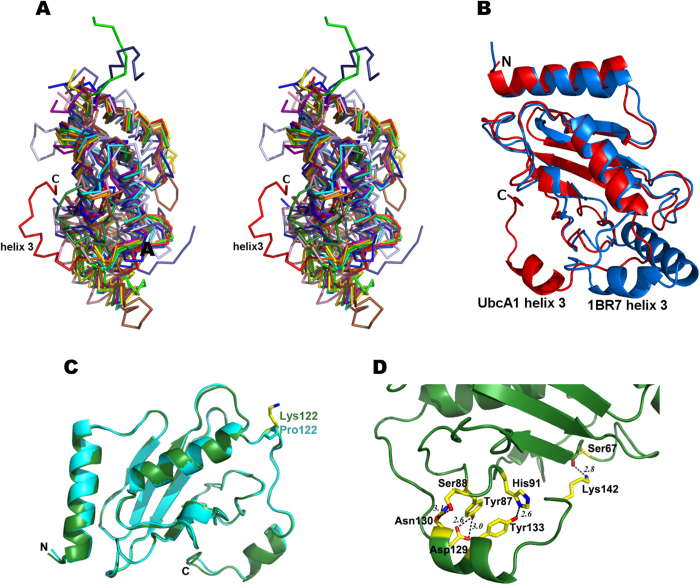
The unique C-terminal conformation of UbcA1. (**A**) Superimposed Cα traces of UbcA1 (red) and 12 representative domains from the Ub-conjugating enzyme superfamily (3.10.110.10) defined in the CATH database^28^, with PDB codes of 1BR7, 1WZV, 1Z2U, 1ZDN, 2GRR, 2YB6, 3K9O, 3H8K, 3CEG, 2A4D, 3OBQ and 1UKX. (**B**) Superimposed ribbon model between UbcA1 (red) and 1BR7 (blue) clearly showing the different orientations of their C-termini. (**C**) The K122P mutant (cyan) shows invariant C-terminal conformation of the wild-type protein (green). (**D**) Hydrogen bonds form around the C-terminal peptide resulting from the intra-subunit interactions. The amino acids involved in hydrogen bond formation are represented by a stick-ball model and colored by element: carbon, yellow; oxygen, red; nitrogen, blue.

The UbcA1 coordinates were sent to the Dali server^29^ for structural comparison with others E2s. Although remarkable structural similarity covered 85% of the amino acid sequence from residue 2 to 121, the C-terminal region (residue 122–142) of UbcA1 unexpectedly did not match any other reported structures. Superimposition of the 12 representative structures from different E2 families classified in CATH onto UbcA1 revealed a distinctive conformation of the C-terminal sequence of this novel Ub-conjugating enzyme (Fig. 6A). These representative domains were taken from the PDB entries of 1BR7, 1WZV, 1Z2U, 1ZDN, 2GRR, 2YB6, 3K9O, 3H8K, 3CEG, 2A4D, 3OBQ and 1UKX. The orientation of the C-terminal helix (α3) of UbcA1 dramatically differed from all those structures, e.g., 1BR7, by almost 180° (Fig. 6B).

The sequence alignments between UbcA1 and other E2s revealed that (i) good conservation existed at the C-terminal sequence after position 122 between UbcA1 and other Ube2w enzymes (Fig. 1A), and (ii) by contrast, the sequence similarity at the same segment between UbcA1 and the 12 representative Ubc domains chosen from CATH was much lower than the overall similarity (Fig. 1B). These comparisons clearly indicate that the C-terminal sequence of E2s is a variant region among different families, but highly conserved within the Ube2w family. We therefore suppose that the unique C-terminal conformation observed in UbcA1 is a family-specific feature. Such a conformation makes helix α3 close to the catalytic Cys residue, which agrees well with the recent NMR structure of Ube2w that displayed a disordered C-terminal region directly beneath the active site^10^.

### The C-terminus is relatively flexible though surrounded by a hydrogen bond network

Given the surprising structural variance observed at the C-terminal peptide between UbcA1 and other reported crystal structures, we next wondered what the structural determinant is for the unique conformation in our structure. The sequence alignment shown in Fig. 1B revealed that a number of E2 sequences from various families contain a proline–rich region (PNPxxP) on the loop between α2 and α3. Compared with these proteins, UbcA1 lacked the first two Pro residues; instead, it contained three consecutive Lys residues at the same positions. Because lysine often behaves as a destabilizer for local conformation by increasing surface entropy, we speculated that these residues rendered the downstream peptide more flexible in this case. To verify the effects of the Lys residues, three mutants (K122P, K124P and K122P/K124P) were produced and crystallized in the same way as the wild-type protein. These structures, however, displayed an unaltered C-terminal conformation (Fig. 6C), suggesting that the amino acid sequence of that loop is not the structural determinant for downstream peptide folding.

In the crystal structure of UbcA1, the C-terminus was exposed on the protein surface and seemed more disordered than other Ub-conjugating enzymes, which was reflected by significantly higher B-factors of the C-terminal region immediately following the second helix (α2) (40.99 from residue 120 to 142) than the rest of UbcA1 (20.253 from residue 2 to 119). Similar flexibility of the C-terminus has also been observed in the NMR structure of Ube2w^10^.

Despite the flexible conformation, the C-terminus of UbcA1 was surrounded by a hydrogen bond network in the crystal (Fig. 6D). Amino acids in helix α3, including Asp129, Asn130, Arg132 and Tyr133, were involved in this network. The hydroxyl of Tyr133 formed a tight hydrogen bond (2.6 Å) with the indole ring of a histidine residue (His91) two positions upstream of the active Cys93, while Asn130 was located within hydrogen bond distance from Ser88 (Fig. 6D). That the C-terminus was oriented so close to the active site may be not only a structural requirement for keeping the unusual C-terminal conformation but also a necessity for remaining the enzymatic activity, as the C-terminus of Ube2w is suggested to facilitate substrate binding^10^. In addition, the loop following helix α3 folded back towards the structural core, which allowed for the formation of a hydrogen bond between Lys142 and Ser67 located on L4.

### The C-terminus significantly affects protein dimerization and E2 activity

Although UbcA1 seemingly forms a dimeric architecture in the crystal, mostly through the interaction between the C-terminal helices in the two neighboring protomers (Fig. 3A), we were not sure whether the C-terminal peptide is sufficient for protein dimerization in solution because human Ube2w also forms a dimer in the crystal, though it was crystallized from a truncated construct without the C-terminal segment after residue 117^27^. To assess the role of this segment, a truncation mutant was made by removing the amino acids downstream of residue 127 (UbcA1-127Δ). The mutant was then subjected to a velocity sedimentation assay using the same protocol as the wild-type protein. The molecular weight of the UbcA1 monomer estimated from sedimentation coefficients was changed from 19.2 to 15.5 Ka upon this truncation, but the dimer signal became less evident, though it did not completely disappear (Fig. 3C), which indicates the monomer-dimer equilibrium that existed in wild-type UbcA1 was greatly abolished in UbcA1-127Δ. This observation disagrees with the SEC elution profile of human Ube2w, which showed almost unaltered equilibrium upon on a similar C-terminal truncation^25^. Our experimental data may imply that the dimerization mechanism probably differs between UbcA1 and Ube2w. UbcA1 seems likely to rely on its C-terminus alone to mediate protein dimerization.

Because UbcA1-127Δ showed weaker tendency to form a dimer, we were curious to determine whether an intact C-terminus is also required for enzymatic activity. The *in vitro* Ub-loading assay was repeated using UbcA1-127Δ instead of the full-length protein. Neither α-NH_2_ nor thiol ubiquitination was observed on either a Coomassie-stained SDS gel or a western blot within a 30-min reaction (Fig. 2C), which indicated that the truncated enzyme completely lost the Ub-conjugating activity. This was another distinct property compared with human Ube2w, in which a C-terminal truncated protein (removal after residue 131) is still capable of forming a thioester intermediate with ubiquitin.

## Discussion

Ubiquitin-conjugating enzymes are central players of the Ub transfer choreography in the ubiquitin-proteasome pathway. The important molecular functions of E2s in the process of Ub chain assembly have been increasingly recognized in recent studies^1^,^2^,^3^,^4^. Nevertheless, structural and mechanistic insights into this superfamily are still insufficient due to high structural diversity among various protein families, which results in different family-specific physiological functions^16^. The available crystal structures do not cover all E2 families, though hundreds of E2 structures have been deposited in PDB^12^. In this study, the crystal structure of a novel Ub-conjugating enzyme was determined. This is the first E2 structure from a fungus other than yeast and the first full-length X-ray structure in the Ube2w family.

The UbcA1 structure reveals a unique conformation of the C-terminal peptide from residue 122 to 142, although its core structure resembles the canonical architecture of the Ubc domain. In this case, helix α3 orients differently from all previously reported E2 structures except the NMR ensemble of Ube2w^10^ (Fig. 6). Such a striking conformation is likely a family-specific feature because the C-terminal sequence of UbcA1 shows good conservation within the Ube2w family (Fig. 1A) but not with Ub-conjugating enzymes from other families (Fig. 1B). Our study further indicates that the C-terminal peptide downstream of residue 127 significantly affects the monomer-dimer equilibrium (Fig. 3B, C), thus showing that this region is an important, if not the determinant, structural element involved in protein dimerization.

In the Ub-loading assay, UbcA1 showed exactly the same activity as Ube2w (Fig. 1A,B). UbcA1 mediates Ub-attachment to the α-NH_2_ group of itself even in the absence of an E3 enzyme. Both enzymes can transfer a single wild-type Ub but multiple HA-Ubs to substrates, suggesting that recognition of backbone atoms could be a common mechanism shared by all members in this protein family. At variance from Ube2w, however, the C-terminal truncation in UbcA1 completely abolishes its Ub-conjugating activity (Fig. 2C), which indicates that the C-terminal region of UbcA1 plays a more crucial role in the enzyme turnover. Despite the high degree of sequence conservation in the C-terminal region throughout the Ube2w family (Fig. 1A) and similar helix α3 positions close to the active site in both the crystal structure of UbcA1 and the NMR ensemble of Ube2w (Fig. 5D), it seems likely that there are subtle differences in the C-terminal region among enzymes in this E2 family, which may result in different roles in the process of Ub-modification. Even so, the unique C-terminal conformation is likely a family-specific feature and serves as a structural determinant of α-NH_2_ ubiquitination.

Some E2s require dimerization for Ub-conjugation^30^,^31^, although most enzymes are monomers under physiological conditions. An intact C-terminus of UbcA1 plays a dual role in both protein dimerization (Fig. 3C) and Ub-loading activity (Fig. 2C). However, we do not know if these two properties are closely linked. A similar truncated version of Ube2w did show much reduced but not 100% loss of enzymatic activity, and almost unaltered monomer-dimer equilibrium^10^,^25^. More studies on this issue are needed to reveal the connection between the physiological function of the enzymes in the Ube2w family and their dimeric state.

## Materials and Methods

### Expression, purification and crystallization

The ORF encoding UbcA1 was inserted into the expression plasmid pET28a (Novagen, Massachusetts, USA) to produce a recombinant protein containing an N-terminal His-tag, which was purified with a two-step chromatography protocol using a Ni-NTA affinity column (15 ml) and a HiLoad 16/60 Superdex75 column (GE Healthcare, Uppsala, Sweden). Crystals used for diffraction data collection were grown under the condition of 20% (w/v) PEG 3350, 0.2 M sodium acetate, and 0.1 M Bis-Tris, pH 6.5.

### Data collection and evaluation

X-ray diffraction data were collected at 100 K under the wavelength of 1.0 Å on beamline BL5A at KEK, Photon Factory, Japan, using an ADSC Q315r CCD detector at a distance of 178 mm with an edge resolution limit at 1.9 Å. The collected diffraction data were indexed, integrated and scaled using *iMosflm*^32^ and *Scala* from the *CCP4* program suite^33^. The scaling statistics showed that the crystal diffracted to even higher resolution than expected. To obtain higher resolution data, integration and scaling were done again with an integration area extending to the half-corner on each image. As a consequence of this evaluation strategy, the data resolution reached 1.7 Å with good I/σ(I) (3.4) but lower completeness (72.2%) in the outermost shell (Table 1). Further details concerning UbcA1 purification, crystallization and data collection are given in another report^34^.

### Structure determination and refinement

The structure of UbcA1 was solved by means of molecular replacement using the *Plasmodium vivax* E2 (PDB code 2FO3) as a search model. Automated structure determination using *Phaser*^35^ and default parameters failed to give apparent solutions, but fortunately two clear solutions were obtained using *AmoRe*^36^, with reasonable correlation coefficients at 56 and R factors at 0.418, indicating two protein monomers sitting in the asymmetric unit. The initial model was manually modified using *O*^37^ and refined using *CNS*^38^ and *Phenix.refine*^39^,^40^. The final model was validated using *Procheck* from the *CCP4* program suite^33^. Statistics from the data collection and structure refinement are summarized in Table 1. Structural comparison and structure-based sequence alignment were performed using the program *STRAP*^41^. All figures representing the UbcA1 structure were generated using the molecular visualization program *PyMol*^42^.

### Mutational analyses

Three single or double site-directed mutants, K122P, K124P and K122P/K124P, as well as a truncated construct named UbcA1-127Δ with deletion of the C-terminal region (residues 128–152), were generated using PCR. All mutants were produced and purified following the same purification protocol as the wild-type protein. The K122P, K124P and K122P/K124P mutants were subsequently crystallized under the same conditions as native UbcA1, and the crystal structures were determined by molecular replacement using the wild-type structure as the search model. The deletion mutant UbcA1-127Δ was subjected to velocity sedimentation assay for oligomeric state detection.

### *In vitro* Ub-loading assay

To assess the activity of UbcA1 to form an α-NH_2_-Ub or thioester-Ub intermediate, an *in vitro* ubiquitin-loading assay was carried out in 30 μl of reaction solution containing 0.5 μM E1 enzyme (human), 40 μM ubiquitin or HA-ubiquitin, 20 μM purified UbcA1 or UbcA1-127Δ, 5 μM MgCl_2_ in 50 mM Tris, 50 mM KCl, pH 7.5. Reactions were initiated by the addition of 5 mM ATP and incubation at 37 °C. A small aliquot of reaction samples was withdrawn at 5, 10, 15 and 30 min after the reaction started. Reactions were stopped by boiling after the addition of SDS sample buffer containing 0 or 200 mM DTT for distinguishing N-terminal Ub-modification and thioester-Ub intermediates. The reaction products were detected by SDS-PAGE using Coomassie blue staining and western blotting using an anti-ubiquitin antibody.

### Ubiquitylation site identification by LC-MS/MS

Protein bands corresponding to the ubiquitylated UbcA1 intermediate shown on a silver-stained SDS gel were excised before reduction (10 mM DTT in 25 mM NH_4_HCO_3_) and alkylation (40 mM iodoacetamide in 25 mM NH_4_HCO_3_) of the cysteines. The gel plugs were digested with trypsin (40 ng for each band), and the resultant sample was analyzed using a Q Exactive LC–MS/MS mass spectrometer (Thermo Fisher Scientific) equipped with an Easy n-LC 1000 HPLC system (Thermo Scientific). Peptides were fractioned on a reverse-phase C18 column (Reprosil-Pur C18 AQ, 3 μm, Dr. Maisch GmbH) with a 78-min gradient elution from 5% to 95% acetonitrile. MS data were collected using a data-dependent top-10 method dynamically choosing the most abundant precursor ions from the survey scan for higher energy collisional dissociation (HCD). Survey scans (m/z 300–1600) were performed at a high resolution of 70,000 at m/z 200, and the resolution for HCD spectra was set to 17,500 at m/z 200. The raw data from Q Exactive were analyzed with Proteome Discovery version 1.4 using the Sequest HT search engine for protein identification and Percolator for the false discovery rate (FDR), against a UniProt human protein database (updated June 2013) containing the Ubca1 protein sequence either with or without an N-terminal extension of GG, corresponding to the last two amino acids of Ub. Two forms of Ubca1 were considered, either containing or omitting the N-terminal methionine residue. FDR analysis was performed using Percolator with 1% FDR set as the criterion for protein/peptide identification.

### Analytical ultracentrifugation

Velocity sedimentation experiments were performed using a 60-Ti rotor equipped in a Beckman XL-I analytical ultracentrifuge at 4 °C, with 12 mm aluminum double-sector cells filled with 400 μL of 40 μM UbcA1 or UbcA1-127Δ dissolved in a buffer containing 50 mM Tris, pH 7.5 and 150 mM NaCl. Raw data were obtained at 280 nm in a continuous scan mode at 0.003 cm intervals with a rotor speed of 60,000 rpm, and analyzed using the c(S) module of *Sedfit*^43^. The buffer parameters, partial-specific volume of the protein, and the corrected sedimentation coefficient S°_20W_ were calculated using *Sednterp*^44^.

## Additional Information

**How to cite this article**: Qi, C. *et al.* Biochemical and structural characterization of a novel ubiquitin-conjugating enzyme E2 from *Agrocybe aegeria* reveals Ube2w family-specific properties. *Sci. Rep.* **5**, 16056; doi: 10.1038/srep16056 (2015).

## Figures and Tables

**Table 1:** Data collection and structure refinement statistics of UbcA1.

Data collection	
Space group	*C2*
Unit cell parameters	
a, b, c (Å)	84.93, 34.76, 128.10
α, β, γ (°)	90, 118.57, 90
Resolution range (Å)*	42.45 - 1.70 (1.79-1.70)
Wilson B-factor	22.5
Multiplicity*	3.4 (2.3)
Completeness (%)*	94.1(72.2)
Mean I / σ (I)*	9.7 (3.4)
Solvent content (%)	41.7
Rsym*	0.044 (0.204)
Structure Refinement	
No. of reflections in working/test set	37002 (3704)
Atoms in model	
No. of non-H protein atoms	2180
No. of water molecules	344
No. of ions	10
R-work/R-free	0.202/0.235
Average of B-factor (Å2)	
Protein	33.4
Solvent	41.4
Ligand/ion	39.5
R.M.S.D.	
R.M.S.D. bond lengths (Å)	0.009
R.M.S.D. bond angles (°)	1.130
R.M.S.D. B-factor for bonded atoms	5.93
MolProbity analysis	
Ramachandran plot	
Most favored region (%)	91.5
Allowed region (%)	8.5
Disallowed region (%)	0
Rotamer outliers (%)	1.65%
C-beta outliers	0
Clashscore	6.90

^*^Values in parentheses are statistics of the highest resolution shell.
